# Supplementary material for: Superadditive and subadditive dynamics are not inherent to the types of interacting threat
Source: PLoS One. 2019 Aug 23;14(8):e0211444. doi: 10.1371/journal.pone.0211444 (PMC6707635; doi:10.1371/journal.pone.0211444)
Supplement: S4 File — (PDF) [file pone.0211444.s004.pdf]

S4 Explanation of data tables

S5 Additivity of random populations across all models

This table includes all of the data necessary to plot figures 2 in the main document and the figures in the supplementary materials. The data is artificial and created using the methods and equations described in the paper, so they can be easily derived again.

The table S5 includes the following variables from column 1-end: Model used (1=logistic, 2=Beverton-Holt, 3=Ricker), combination of threats (1=additive, 2=multiplicative), growth rate (r), carrying capacity (K), harvest rate (h), impact of threat 1 on growth rate (a_1), impact of threat 2 on growth rate (a_2), impact of threat 1 on carrying capacity (b_1), impact of threat 2 on carrying capacity (b_2) and the resulting additivity (A).

S6 Management benefit

This table includes all of the data necessary to plot figures 4 and calculate table 2 in the main document and the figures in the supplementary materials. The data is artificial and created using the methods and equations described in the paper, so they can be easily derived again.

Both tables can be found here

https://figshare.com/articles/Data_for_paper_Superadditive_and_subadditive_dynamics_are_not_inherent_to_the_types_of_interacting_threat_/9253379
